# Cerebral Changes Occurring in Arginase and Dimethylarginine Dimethylaminohydrolase (DDAH) in a Rat Model of Sleeping Sickness

**DOI:** 10.1371/journal.pone.0016891

**Published:** 2011-03-09

**Authors:** Donia Amrouni, Anne Meiller, Sabine Gautier-Sauvigné, Monique Piraud, Bernard Bouteille, Philippe Vincendeau, Alain Buguet, Raymond Cespuglio

**Affiliations:** 1 Université Claude Bernard Lyon 1, Université de Lyon, Faculté de Médecine, EA 4170 and Plateau NeuroChem, Lyon, France; 2 Laboratoire des Maladies Héréditaires du Métabolisme, Centre de Biologie Est, Hospices Civils de Lyon, Lyon, France; 3 Université de Limoges, Faculté de Médecine, EA 3174 and IFR 145 GEIST, Limoges, France; 4 UMR 177 IRD-CIRAD-Université de Bordeaux 2, Bordeaux, France; Texas A&M University, United States of America

## Abstract

**Background:**

Involvement of nitric oxide (NO) in the pathophysiology of human African trypanosomiasis (HAT) was analyzed in a HAT animal model (rat infected with *Trypanosoma brucei brucei)*. With this model, it was previously reported that trypanosomes were capable of limiting trypanocidal properties carried by NO by decreasing its blood concentration. It was also observed that brain NO concentration, contrary to blood, increases throughout the infection process. The present approach analyses the brain impairments occurring in the regulations exerted by arginase and N^G^, N^G^–dimethylarginine dimethylaminohydrolase (DDAH) on NO Synthases (NOS). In this respect: (i) cerebral enzymatic activities, mRNA and protein expression of arginase and DDAH were determined; (ii) immunohistochemical distribution and morphometric parameters of cells expressing DDAH-1 and DDAH-2 isoforms were examined within the diencephalon; (iii) amino acid profiles relating to NOS/arginase/DDAH pathways were established.

**Methodology/Principal Findings:**

Arginase and DDAH activities together with mRNA (RT-PCR) and protein (western-blot) expressions were determined in diencephalic brain structures of healthy or infected rats at various days post-infection (D5, D10, D16, D22). While arginase activity remained constant, that of DDAH increased at D10 (+65%) and D16 (+51%) in agreement with western-blot and amino acids data (liquid chromatography tandem-mass spectrometry). Only DDAH-2 isoform appeared to be up-regulated at the transcriptional level throughout the infection process. Immunohistochemical staining further revealed that DDAH-1 and DDAH-2 are contained within interneurons and neurons, respectively.

**Conclusion/Significance:**

In the brain of infected animals, the lack of change observed in arginase activity indicates that polyamine production is not enhanced. Increases in DDAH-2 isoform may contribute to the overproduction of NO. These changes are at variance with those reported in the periphery. As a whole, the above processes may ensure additive protection against trypanosome entry into the brain, i.e., maintenance of NO trypanocidal pressure and limitation of polyamine production, necessary for trypanosome growth.

## Introduction

Human African trypanosomiasis (HAT), or sleeping sickness, is caused by the protozoan parasites *Trypanosoma brucei (T. b.) gambiense* or *T. b. rhodesiense*. These parasites, transmitted by the bite of an infected tse tse fly, cause a two-stage disease. The early hemolymphatic stage 1 corresponds to the proliferation of the parasite in the blood and lymph. This stage is followed by the meningoencephalitic stage 2 during which the parasites invade the central nervous system (CNS) [1]. If untreated, the disease evolves towards death in a deep cachectic condition.

**Figure 1:**
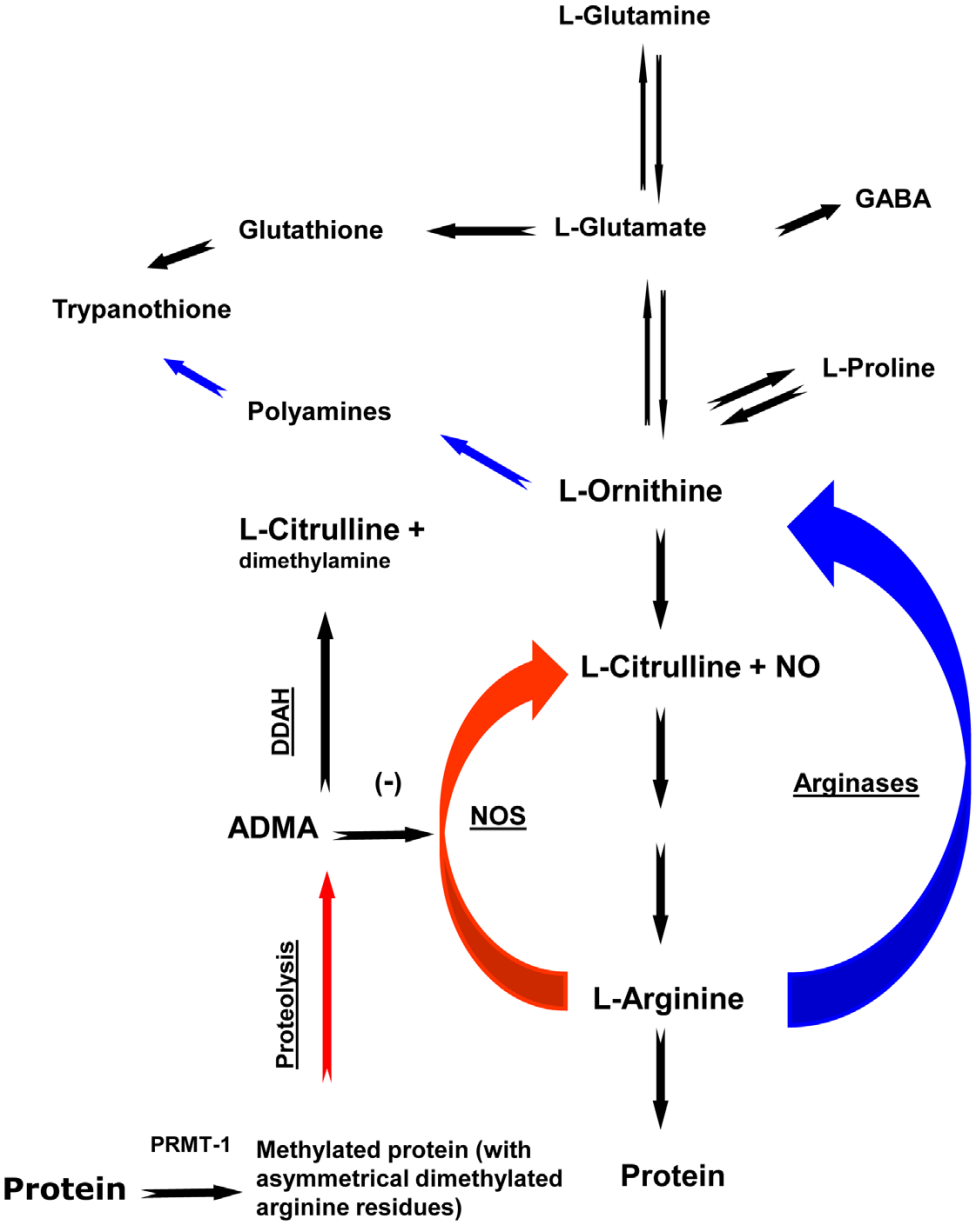
Simplified scheme of L-arginine pathways in mammalian host cells. L-arginine is a crossroad for multiple pathways since it is the common substrate of arginase and NOS that catalyzes the production of L-ornithine, L-citrulline and NO, respectively. ADMA, a product of the proteolysis of methylated proteins by PRMT-1, is a potent inhibitor of NOS. Its concentration is regulated by DDAH that catalyzes the production of L-citrulline and dimethylamine. L-ornithine and L-glutamate serve as precursors of polyamines and glutathione. These two metabolites will be further utilized by the trypanosomes for the synthesis of trypanothione, an essential element protecting the parasites from free radicals. Abbreviations: NO, nitric oxide; NOS, nitric oxide synthase; ADMA, asymmetric dimethylarginine; DDAH, N^G^,N^G^-dimethylarginine dimethylaminohydrolase; PRMT-1, Protein arginine N-methyltransferase 1; GABA, γ-aminobutyric acid.

Nitric oxide (NO), a gaseous signaling molecule that acts as a messenger in the immune and nervous systems, has been involved in the physiopathological processes that occur in the rat model of HAT [2]. NO is a highly reactive molecule possessing a very short half-life (<0.5 second) and is synthesized from the amino acid L-arginine by three major isoforms of NO Synthase (NOS): the neuronal NOS (nNOS, type 1), the inducible NOS (iNOS, type 2) and the endothelial NOS (eNOS, type 3) [3]. The production of NO by iNOS takes place during inflammatory and infectious situations. Accordingly, it is a key element in counteracting the proliferation of trypanosomes through its cytotoxic properties [4], [5]. The biological availability of NO is achieved directly by the activity of the NOS enzymes. These enzymes are further submitted to a complex regulation involving arginase and N^G^, N^G^-dimethylarginine dimethylaminohydrolase (DDAH) enzymes (Fig. 1). Arginase, in catalyzing the last step of the urea cycle, leads to the production of L-ornithine, a synthesis precursor of L-proline, L-glutamate, L-glutamine, γ-aminobutyric acid (GABA) and polyamines (Fig. 1). Arginase exists in two isoforms: (i) arginase-1 (liver isoform) is strongly expressed in the cytosol of liver cells; (ii) while arginase-2 (extra-liver isoform, predominant in kidney) is localized into the mitochondrial membrane of cells. In the brain, arginase-1 and, to a lesser extent, arginase-2 have been identified in neurons but not in glial cells.

Arginase activity is particularly high in brain regions with strong nNOS expression [6]. Arginase may contribute to reduce NOS activity, due to the use of the common substrate, L-arginine. Conversely, arginase may be inhibited by N-hydroxy-L-arginine, the intermediate product of the reaction catalyzed by NOS [7]. Another potent way regulating NO synthesis is mediated by DDAH. This enzyme catalyzes the hydrolysis of asymmetric dimethylarginine (ADMA) to dimethylamine and L-citrulline. ADMA is a product of the catabolism of arginine-methylated proteins by protein arginine N-methyltransferase 1 (PRMT-1) [8] (Fig. 1). As for arginase, two isoforms of DDAH have been described, DDAH-1 and DDAH-2. It is reported that DDAH-1 is typically located in nNOS-containing tissues, whereas DDAH-2 predominates in iNOS-expressing tissues and in vascularized areas where eNOS is present [9]. All NOS isoforms can be inhibited by ADMA, the substrate of DDAH. Under certain conditions, the S-nitrosylation of DDAH by NO may lead to a strong decrease in its activity, leading subsequently to an accumulation of ADMA and NOS inhibition. This feedback mechanism may thus contribute to the regulation of NO production [10].

In a recent study conducted in the rat infected with *T. b. brucei*, an experimental model of HAT, the production of NO was analyzed both in the brain (hypothalamus/thalamus) and the periphery (blood) [2]. Changes occurring in NO production were dependent on iNOS activity. In the periphery, two main observations were recorded: (i) a marked decrease in blood NO production which likely limits the NO trypanocidal activity; and (ii) an increased activity of arginase favouring the synthesis of polyamines necessary for trypanosome growth [11]. In the brain, on the contrary, an increase in NO production occurred due to an enhanced iNOS activity in neurons and glial cells. Consequently, the iNOS activation observed in brain cells was primarily considered as a marker of deleterious inflammatory processes.

To date, the brain mechanisms of this infection are part of the most severe aspects of the disease and remain unknown. The present work, an extension of the above investigations [2], focused on the relative role of arginase and DDAH in the brain during the different stages of *T. b. brucei* infection. Measures were performed throughout the time course of the infection in the rat model infected with *T. b. brucei* to assess (i) the cerebral changes occurring in the enzymatic activities, mRNA and protein expression of arginase and DDAH; (ii) the determination of the immunohistochemical distribution and the morphometric parameters of the cells expressing the isoforms of DDAH (DDAH-1 and DDAH-2) within the diencephalon; and (iii) the changes occurring in the profile of the amino acids related to the NOS/arginase/DDAH pathways.

## Materials and Methods

### Ethics statement

All animals were handled in strict accordance with good animal practice in compliance with the relevant decree of the French Agriculture Ministry (N°: 03-505). Animal procedure was approved by Departmental Direction of Veterinary Services of Rhône-Alpes.

### Animals and infection

Male Wistar rats (Janvier breeding, Le Genest Saint Isle, France) weighing 200–220 g, were kept under standard laboratory conditions: 12 h/12 h light/dark cycle, ambient temperature of 21 to 22°C, food and water *ad libitum*. After one week of adaptation, two groups of animals (control, n = 24; infected, n = 24) were constituted. The animals were infected with the AnTat 1.1 E clone of *T. b. brucei* (Institute of Tropical Medicine, Antwerp, Belgium). Before infection, the mobility and viability of trypanosomes were controlled under microscope. The intraperitoneal (i.p.) injection of 3,600 parasites defined the initial day (D0) of the experimental session. In parallel, control animals received an i.p. injection of the same volume of physiological saline solution.

In the infected rats (HAT experimental model), body weight was measured and blood parasites counted every two days as previously described [2]. Cerebrospinal fluid (CSF) samples were obtained from *cisterna magna* puncture achieved under chloral hydrate anesthesia (400 mg/kg). Parasite counts in CSF samples were performed every four days after infection (from D10 to D22). The biological signs characterizing the entry into the neurological state of the disease appeared, as previously described [2], from D5 to D10 post-infection. At this time, however, the parasites were not yet observed in the CSF (hemolymphatic stage 1). From D10 to D22, while the body weight gain continue to declines, trypanosomes were observed in the CSF (neurological stage 2). The animals were sacrificed by decapitation at days D5, D10, D16 and D22 post-infection.

### Dissection and protein extracts from brain tissue

In order to obtain protein extracts from cerebral tissues, the brain of each animal was quickly removed, briefly rinsed in ice-cold Tris-EDTA buffer (50 mM, pH 7.4), and dissected to collect the diencephalic (hypothalamus/thalamus) structure. Protein extracts from brain tissue were obtained as previously described [2] and protein concentrations were determined in supernatant fractions using Bradford's method [12].

### Plasma samples

Blood samples were obtained from direct puncture of the heart with a heparinized syringe and centrifuged at 2,400 g (4°C, 10 min). Then, plasma supernatant was removed and samples kept at −80°C until use.

### Arginase and DDAH activity in brain tissue

#### Measurement of arginase activity

Arginase activity was determined by analyzing the conversion of L-arginine to urea with the colorimetric method described by Liu et al. [13]. Briefly, for the enzyme activation, 15 µL of a solution containing 10 mM MnCl_2_ in 50 mM Tris-HCl (pH 7.5) were added to 15 µL of each diencephalic protein extract (about 75 µg of proteins). The mixture was incubated at 55°C for 10 min. Then, 30 µL of the substrate, 0.2 M L-arginine, were added and the new mixture incubated for 5 min at 37°C. The enzymatic reaction was stopped by adding 40 µL of 20% H_2_SO_4_. To quantify urea formation, 2.5 mL of a 20% H_2_SO_4_ solution containing 0.4% w/v antipyrin, 0.005% w/v iron (III)-sulfate hydrate and 2.5 mL of a 5% acetic acid solution containing 0.5 w/v diacethyl monoxim were added. After incubation at 100°C for 15 min, the absorbance of the colored product was measured at 450 nm. All assays were carried out in duplicates. The blank assay was performed in the same way, except that the enzymatic reaction was stopped with the acidic solution immediately after addition of the substrate to each diencephalic protein sample. The specific activity corresponding to nanomoles of urea formed per min and per mg of proteins (nmol/min/mg) was expressed as % of the values obtained in the non-infected condition. The arginase activity obtained from samples of rat liver was used as positive control.

#### Measurement of DDAH activity

DDAH activity was measured by analyzing the conversion of ADMA to L-citrulline. After the addition of 225 µL of 5 mM ADMA in 100 mM phosphate buffer (pH 6.5) to 25 µL of each diencephalic protein extract (about 125 µg of proteins), the mixture was incubated at 37°C for two hours. The reaction was stopped by the addition of 250 µL of 10% tricloroacetic acid (ice-cold). After centrifugation (10 min at 3,000 g), 250 µl of a 20% H_2_SO_4_ solution containing 0.5% w/v antipyrin and 250 µl of a 5% acetic acid solution containing 0.8% w/v diacethyl monoxim, were added to each supernatant sample (about 490 µL) containing L-citrulline. The mixtures were then incubated at 100°C for 12 min before measuring the absorbance at 466 nm as described by Prescott and Jones [14]. All assays were carried out in duplicate. The blank assay was performed in the same way, except that the enzymatic reaction was immediately stopped (by the ice-cold tricloroacetic acid) just after addition of the buffered substrate solution to each diencephalic protein sample. The specific activities (picomoles of citrulline formed per min and per mg of protein (pmol/min/mg)) were expressed in % of the values obtained in the non-infected condition. The DDAH activity obtained from samples of rat kidney was used as positive control.

### Western-blotting

Forty µg of the total protein extract obtained from diencephalic samples was loaded and separated on NuPAGE® Novex® 10% Bis-Tris gels with MOPS running buffer (Invitrogen-Life Technologies, Cergy-Pontoise, France). Protein transfers to nitrocellulose membranes were performed using the iBlot™ DryBlotting device (Invitrogen) and iBlot™ Transfer stacks (program P3 for 7 min). After transfer, membranes were first saturated for one hour at room temperature in buffer A (Tris 50 mM pH 7.5, NaCl 166 mM, Tween 0.2%) containing 5% of non fat dry milk, followed by an incubation with the rabbit polyclonal anti-DDAH-1 (1/500, Orbigen, San Diego, USA), rabbit polyclonal anti-DDAH-2 (H-85 1/200, Santa Cruz Biotechnology, California, USA), rabbit polyclonal anti-arginase-1 (H-52, 1/200, Santa Cruz Biotechnology), rabbit polyclonal anti-arginase-2 (H-64, 1/200, Santa Cruz Biotechnology) or mouse anti-β actin antibody (1/1000, Santa Cruz Biotechnology) for 6 hours in buffer A containing 2.5% of non fat dry milk. After three washings in buffer A, membranes were incubated during one hour with corresponding biotinylated secondary antibody, goat anti-rabbit (1/2000, Vector Labs, ABCYS, Paris, France), or goat-anti mouse (1/1000, Vector Labs) in buffer A containing 2.5% non fat dry milk, washed again in buffer A, then incubated for two hours with avidin-peroxidase (1/1000, Vectastain kit, Vector Labs) in buffer A. Blots were then washed three times and the conjugates were visualized by enhanced chemiluminescence (ECL) (Super Signal West Pico, Pierce, Thermo Scientific, and Courtabeuf, France). The ECL signals were acquired with ChemidocXRS (BioRad, Marnes-la-Coquette, France) and quantified with Quantity One software (BioRad). Blots of anti-β actin were used as internal standard and samples of kidney and liver as positive controls. Quantification of the blots was performed using a BioRad ChemiDoc software. Mean values determined are given for control and experimental groups at different days post infection (D5, D10, D16 and D22).

### Real-time RT-PCR

As previously described [15], total RNA was prepared from diencephalic brain samples using a RNA-plus solution (Q-Biogen, Illkirch, France). For each gene, the sequence of the primer pairs and the annealing temperature are given in Table 1. Results were normalized to 18S rRNA as endogenous control and expressed in percent of the corresponding values of the non-infected condition.

**Table 1:** Real-time PCR analysis: sequence of the oligonucleotide pairs, annealing temperature (Ta) and product size.

Genes	Primer pairs	Ta (°C)	Product size (bp)
**DDAH-1**	ACTCACTCCAGCCTCTGTGT TCCACGTTCTCAGGACTCTC	57	153
**DDAH-2**	AACTGAGGCAACGACTAGGT GATTAGAGCCGTGTCTCCTT	58	113
**ARG-1**	GGCAGTGCCGTTGACCTTGT AGCAGCGTTGGCCTGGTTCT	59	158
**ARG-2**	TGTCGGCTCTGGATCTTGTT AATGTGTCCGCCTTCTCTTG	57	131
**18S**	TAGAGGGACAAGTGGCGTTC CGCTGAGCCAGTCAGTGTAG	64	100

### Immunohistochemistry of DDAH-1 and DDAH-2

Animal perfusion, brain fixation and cutting were achieved as previously described [2]. Saturation of the non specific binding sites of the antibody was performed by incubating the brain sections (25 µm thick) for 90 min in a phosphate buffered saline solution (PBS; 50 mM, pH 7.4) containing 1% of bovine serum albumin (BSA). Rabbit polyclonal anti-DDAH-1 antibody (1/500, Orbigen, San Diego, USA) and goat polyclonal anti-DDAH-2 antibody (1/500, Abcam, Paris, France) were used as primary antibody (2×24 h at 4°C). Biotinylated goat anti-rabbit (1/500, Vector Labs, ABCYS, Paris, France) and rabbit anti-goat (1/500, Vector Labs) were used as secondary antibody (12 h at 4°C). Three infected or un-infected rats were used for DDAH-1 and DDAH-2 immunohistochemistry. Rat kidney, expressing predominantly DDAH isoforms, was used as specific controls for the different antibodies employed.

### Morphometric analysis of DDAH-1 and DDAH-2 expressing cells

The analysis was achieved on the ventral and posterior parts of the thalamus, an area where DDAH-1 and DDAH-2 immunoreactivity had been observed. The morphometric parameters employed were as follows: (1) Soma area (µm^2^); (2) Soma perimeter (µm); (3) Soma form factor (FF* = 4π×Area/Perimeter, FF* values equaled 1.0 for a perfect circle and <1.0 for all ellipses).

Measures were taken from 268 elements, i.e., 134 cells for each DDAH brain section isoform (DDAH-1 or DDAH-2). The external border of the soma was delimited by way of a computer mouse, then, the area of the soma, perimeter and form factor were automatically calculated by the Mercator software which was related to the Axioscope2 plus microscope and the QIMAGING camera.

### Amino acids (AA) assay by liquid chromatography tandem-mass spectrometry (LC-MS/MS)

L-Arginine, L-glutamate, L-glutamine, L-proline and GABA were quantified in diencephalic brain structures as underivatized AA by the LC-MS/MS method previously described by Piraud et al. [16]. Only L-Arginine, L-glutamate and L-glutamine were quantified in blood samples by the same method. L-Citrulline, L-ornithine (in brain) and ADMA (in blood), were quantified as butyl esters derivatives in order to improve the detection limit. The method used is adapted from Schwedhelm et al. [17] with some slight modifications of the chromatographic gradient and the internal standard.

### Derivatized AA assay

#### Standards and samples preparation

Fifty µL of calibration standard solutions (L-citrulline, L-ornithine or ADMA in increasing quantities: 0, 0.5, 1, 2, 5, 10, and 20 µM each), or 50 µL of brains extracts or 50 µL of plasma samples, were mixed with 20 µL of internal standard solution at 14 µM L-[^2^H] proline (Pro*), L-[^2^H] ornithine (Orn*) and L-[^2^H] arginine (Arg*) (Cambridge Isotope Laboratories, Andover, USA). Protein and lipid precipitation was performed by adding 400 µL of ethanol and 400 µL hexane to all samples. The samples were then mixed for 2 min and stored for 10 min at room temperature. Afterwards, the supernatant was eliminated and the residual phase again precipitated by 400 µL of hexane. After centrifugation during 5 min at 17,500 g, dried supernatant were derivatized by butylation using 200 µL of 3N HCl in butanol (Interchim, Montluçon, France), followed by evaporation according to Schwedhelm et al. [17]. Finally, dried samples were reconstituted in 50 µL of the mobile phase (formic acid/methanol, 1 mL/1 L) and fractions of 5 µL of brain extracts or 20 µL of plasma were injected into the LC apparatus.

#### LC and gradient conditions

The derivatized AA separation was achieved using reversed-phase liquid chromatography (Waters Alliance 2795 LC set-up, Waters Corporation, Beverly, MA, USA) with a Varian analytical column (2.0×50 mm) packed with a Polaris C18-Ether (3 µm Bead size, Varian, Courtaboeuf France). Before use, the column was rinsed with methanol during one hour at a flow rate of 0.1 mL/min, then, overnight, with methanol/water (50/50, v/v). The chromatography was performed at 25°C with a flow rate of 0.2 mL/min. The gradient started with 10% mobile phase A (formic acid/methanol, 1 mL/1 L) and 90% mobile phase B (formic acid/water, 1 mL/1 L), then increased linearly during 2 min until 50% A, which is maintained during 5 min, and then back to 10% A during 5.5 min. Afterwards, 10% A was maintained during 15 min in order to re-equilibrate the column before a new injection. The LC set-up was connected to the electrospray source for ionization (ESI) of the tandem mass spectrometry.

#### MS/MS

The AA identification was carried out on a Quattro micro triple quadruple tandem mass spectrometer (Waters Corporation). The electrospray source was used in positive ionization mode for all analyses. Nitrogen and argon were used as drying and collision gases, respectively. Source temperature was fixed at 120°C and desolvatation temperature at 350°C. The cone gas flow was fixed at 60 L/h, the desolvatation gas flow at 600 L/h, the voltage multiplier at 650 V and the dwell time at 0.05 s. All results were acquired with the MassLynx software version 4.0. Specific MS, LC parameters (cone voltage, CV in V; collision energy, CE in eV; retention time, RT in min) and internal standards (AA*) used are given in Table 2.

**Table 2:** Specific parameters used for identification of amino acid in MS/MS positive ion mode.

	Monitored transition (m/z)	CV (V)	CE (eV)	AA*	RT (min)
***Derivatized (butylated) AA***					
L-citrulline	232>113	20	18	L-pro*	3.11
L-ornthine	189>70	12	18	L-orn*	1.10
ADMA	259>214	28	14	L-arg*	2.16
***Underivatized AA***					
L-arginine	175>70	20	32	L-arg*	12.71
L-proline	116>70	20	30	L-pro*	2.38
L-glutamate	148>84	15	24	L-glu*	2.07
L-glutamine	147>84	20	24	L-gln*	2.02
GABA	104>87	20	14	L-lys*	9.16

AA: Amino acid.

CV, CE correspond to the cone voltage (in V), and collision energy (in eV), respectively.

AA* corresponds to the stable labelled amino acid (AA) chosen as internal standard for the quantification.

RT corresponds to the time of retention (in min) of each analyte under selected chromatographic conditions.

ADMA: asymmetric dimethylarginine. GABA: γ-aminobutyric acid.

### Underivatized AA assay

The quantitative determination of underivatized AA was achieved as reported by Piraud et al. [16]. All MS, LC parameters and internal standards (AA*) used for each AA are given in Table 2.

### Statistical Analysis

Results are expressed as the mean ± standard error of the mean (SEM). Statistical analysis of the differences between rat groups (n = 6 animals in each group) at the different times post- infection (D5, D10, D16, D22) was performed by one-way analysis of variance (ANOVA). When ANOVA was significant at p<0.05, a post hoc Fisher's least significant differences test was applied.

## Results

### Changes occurring in DDAH and arginase brain activity

**Figure 2:**
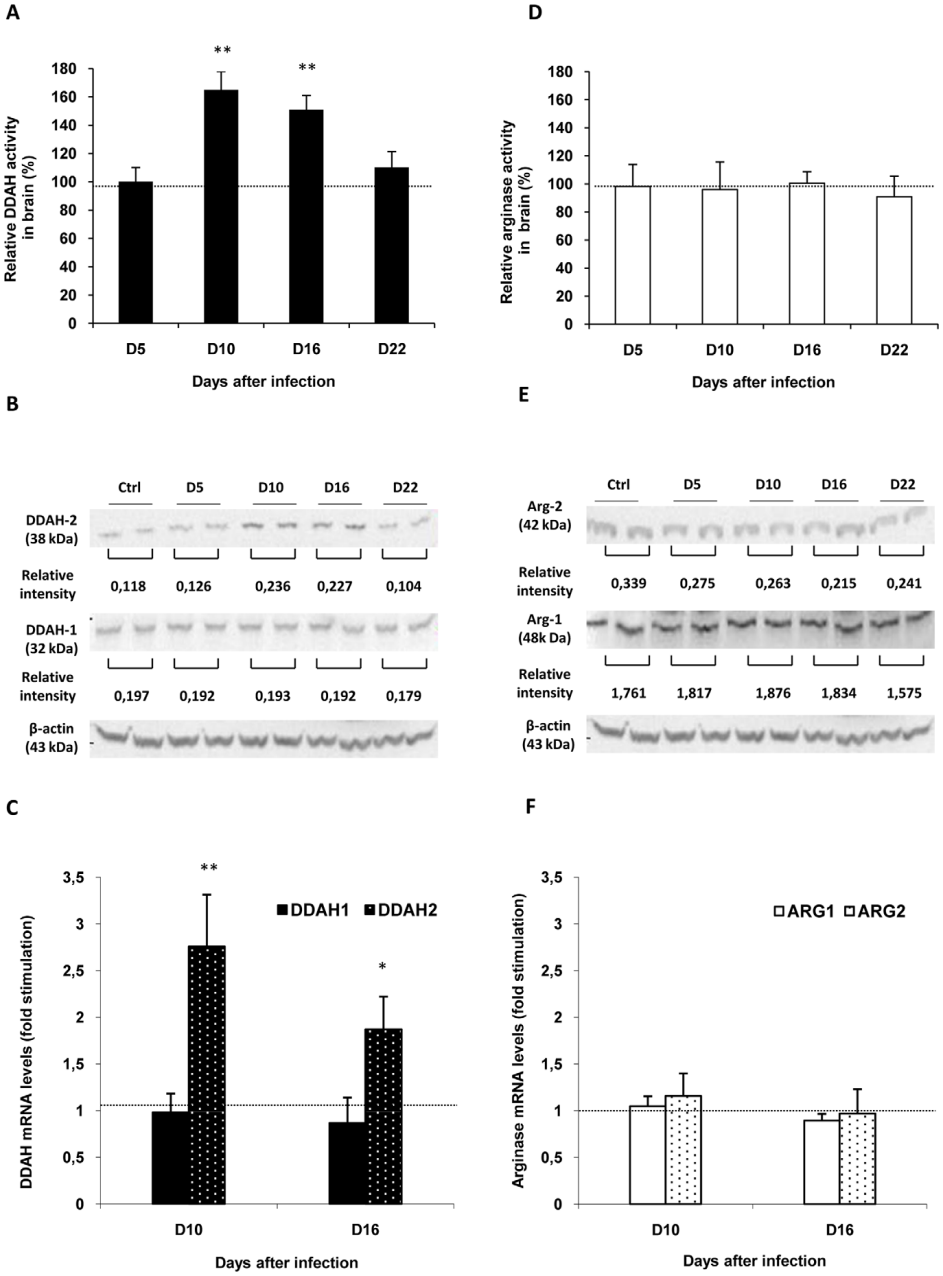
Relative changes in activity, mRNA and protein expression of DDAH and arginase in the brain during the course of infection in *Trypanosoma brucei brucei* (*T. b. b.*) infected rats. DDAH (A) and arginase (D) activities were measured in diencephalic (hypothalamus/thalamus) biopsies. Results (mean value ± SEM, n = 6 per experimental day) were normalized with corresponding values measured in control rats (n = 6 per experimental day). Control values (100%), delineated by the dotted line, correspond to 269.66±7.09 pmol/min/mg for DDAH and to 26.34±0.40 pmol/min/mg for arginase. Relative intensity of DDAH (B) and arginase (E) bands in western blot was calculated for each band and the results expressed as a mean value for control and different experimental days (β-actin was used as an internal standard). The significant up-regulation taking place for *DDAH-2* transcription (C) is not observed for *DDAH-1*(C), *ARG-1* or *ARG-2 (F)*. The transcript levels for *DDAH-1*, *DDAH-2*, *ARG-1 and ARG-2* were estimated by real-time quantitative RT-PCR and normalized to 18S rRNA as endogenous control. The graphs represent the mean ± SEM of 4 independent experiments (n = 4 per experimental day). Statistics: ANOVA followed by post-hoc Fisher's test (*p<0.05, **p<0.001 compared to healthy rats). Abbreviations: DDAH, N^G^, N^G^-dimethylarginine dimethylaminohydrolase; *DDAH-1*, DDAH isoform 1; *DDAH-2*, DDAH isoform 2; Ctrl: Control; *ARG-1*, arginase isoform 1; *ARG-2*, arginase isoform 2.

Compared to healthy control rats, a significant increase in DDAH activity was observed at D10 (164.81±13.11%) and D16 (150.89±10.01%) in diencephalic brain structures. Afterwards, at D22 post-infection, the activity decreased towards control values (Fig. 2A). No significant variation was observed in arginase activity throughout the time course of the infection (Fig. 2D).

### Brain expression of the two isoforms of DDAH

#### Accumulation of DDAH-2 protein isoform in the brain of infected animals

Western blot analysis of hypothalamic and thalamic tissue confirms the presence of DDAH-1 and DDAH-2 in these brain structures (Fig. 2B). In agreement with data on DDAH activity, DDAH-2 protein levels increase in infected animals at D10 and D16 post-infection (Fig. 2B). Therefore, DDAH-1 did not differ between the two animal groups throughout the time course of the infection (Fig. 2B).

#### Up-regulation of DDAH-2 transcription in the brain of infected animals

Compared to healthy control animals, a significant increase in mRNA transcripts of DDAH-2 was observed in the diencephalon of infected animals at D10 (2.76±0.64 fold) and D16 (1.87±0.64 fold) (Fig. 2C). No change was observed in the mRNA transcripts of DDAH-1.

#### Brain distribution of DDAH-1 and DDAH-2 protein isoforms

**Figure 3:**
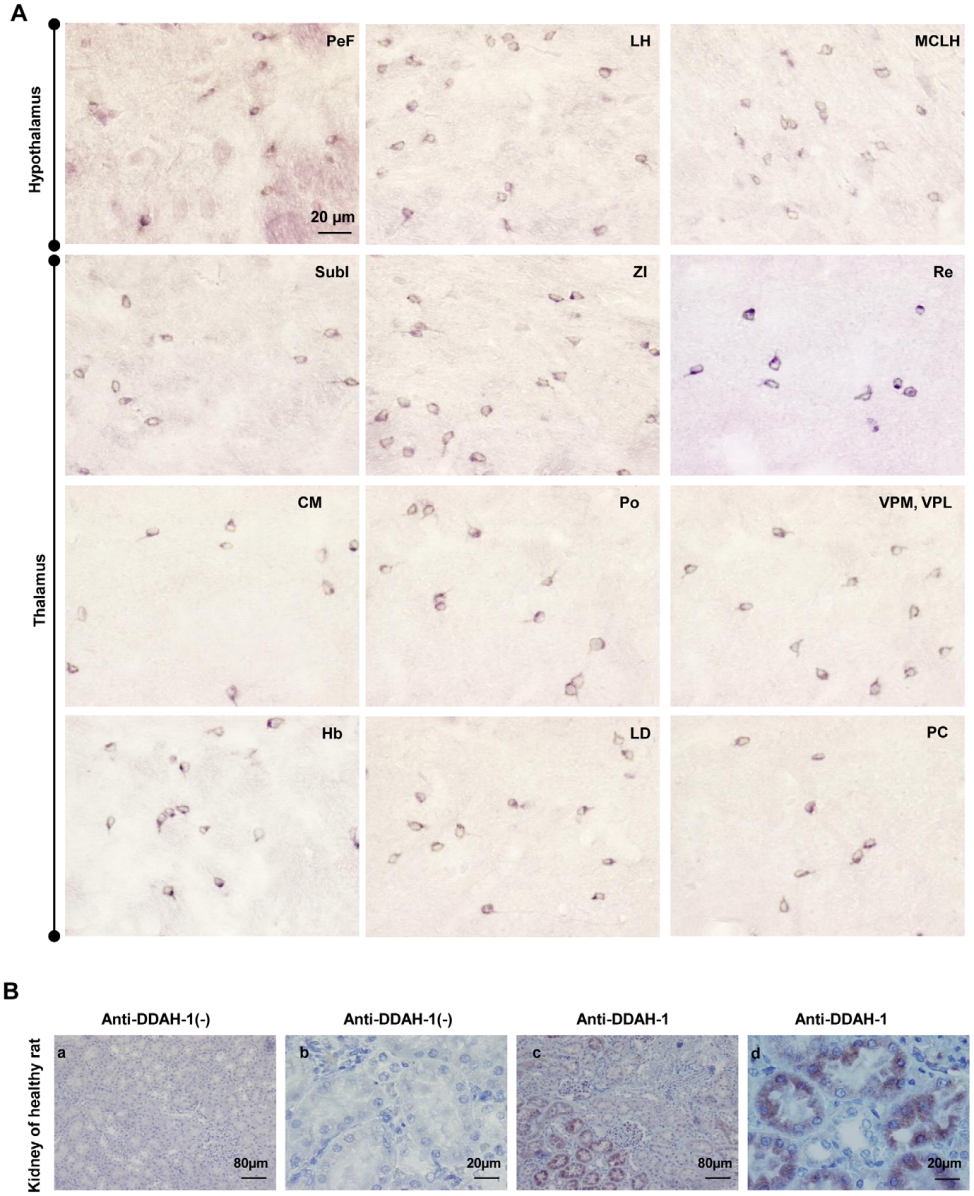
Immunostaining of DDAH-1 in the diencephalon of *T. b. b.-*infected rats. (A) Immunoreactivity of DDAH-1 observed in different hypothalamic and thalamic structures: perifornical nucleus (PeF), magnocellular nucleus of the lateral hypothalamus (MCLH); lateral hypothalamic area (LH); subincertal nucleus (SubI); zona incerta (ZI); reuniens thalamic nucleus (Re); central medial thalamic nucleus (CM); posterior thalamic nuclear group (Po); ventral posterolateral (VPL) and posteromedial (VPM) thalamic nucleus; paracentral thalamic nucleus (PC); laterodorsal thalamic nucleus (LD); and habenular nucleus (Hb). (B) Immunostaining of DDAH-1 (B-b, d) in kidney of healthy rats (control of specificity for anti-DDAH-1). For control for the specificity of the secondary antibody, kidney sections were incubated without anti-DDAH-1 (B-a, c). Number of animals, n = 3. The microscope used was a Nikon Eclipse E400 equipped with Sony DXC-390P camera (objective: X20, X40). Abbreviations: DDAH-1, N^G^, N^G^-dimethylarginine dimethylaminohydrolase isoform 1; see also figure 2.

DDAH-1 and, to a lesser extent, DDAH-2 immunoreactivities were observed in several brain areas (cortex, hippocampus, amygdala) and notably in the hypothalamic and thalamic groups of nuclei in healthy and infected rat (at D10 and D16 post-infection) groups. For DDAH-1, a cytosolic immunoreactivity was clearly identified among the interneurons of the perifornical (PeF) nucleus and the magnocellular part of the lateral hypothalamus. DDAH-1 immunoreactivity was also observed in the zona incerta (ZI), the habenular nuclei (Hb) and subincertal (SubI), reuniens (Re), central medial (CM), posterior (Po), ventral posterolateral (VPL), posteromedial (VPM), paracentral (PC), and laterodorsal (LD) thalamic nuclei (Fig. 3A).

**Figure 4:**
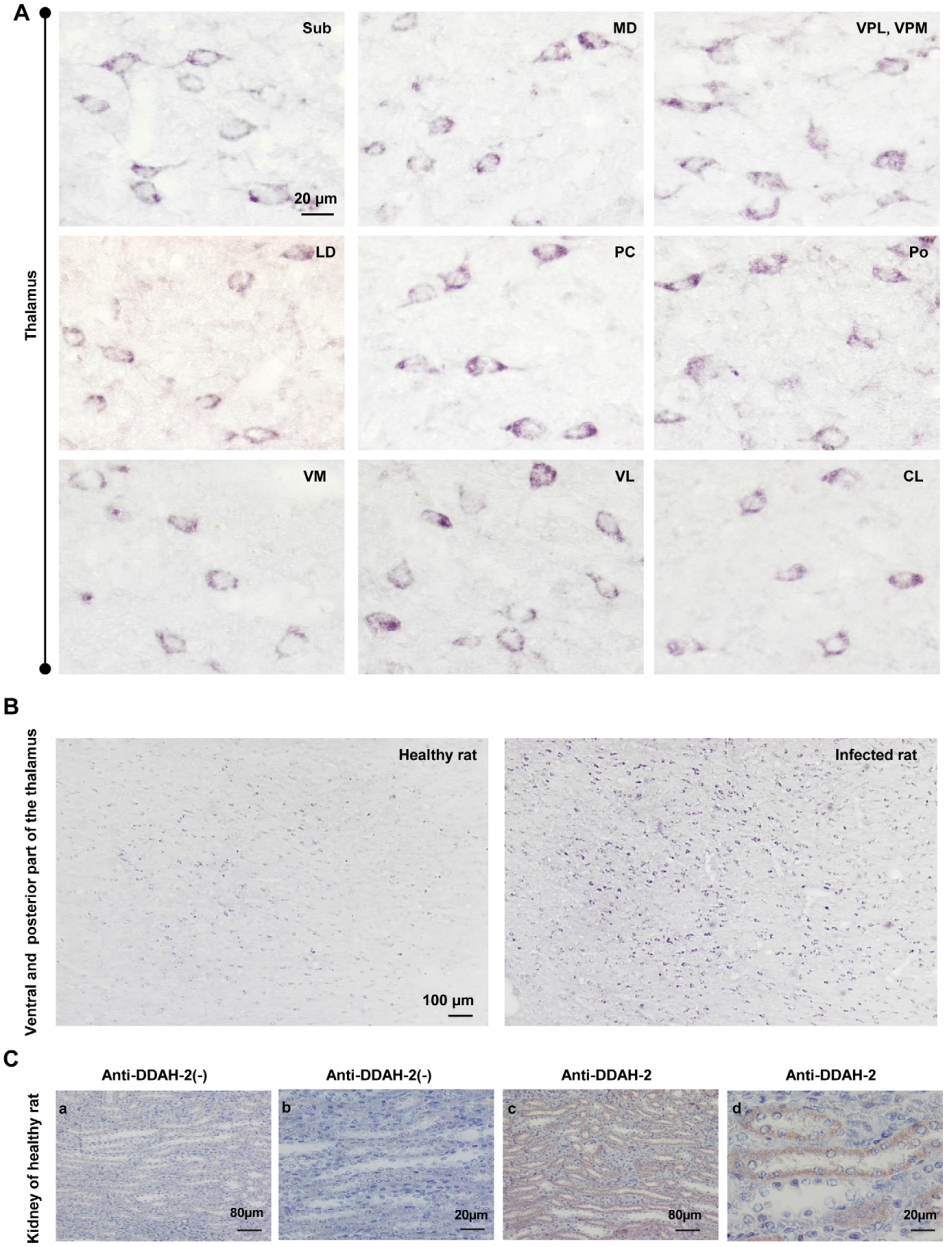
Immunostaining of DDAH-2 in the diencephalon of *T. b. b.-*infected rats. (A) Immunohistochemistry for DDAH-2 observed in different thalamic structures: submedius thalamic nucleus (Sub); mediodorsal thalamic nucleus (MD); ventral posterolateral and posteromedial thalamic nucleus (VPL, VPM); laterodorsal thalamic nucleus (LD); paracentral thalamic nucleus (PC); posterior thalamic nuclear group (Po); ventromedial thalamic nucleus (VM); ventrolateral thalamic nucleus (VL); centrolateral thalamic nucleus (CL). (B) Comparison of the DDAH-2 protein expression shown by immunohistochemistry in the ventral posterior nucleus of the thalamus between infected animals and control healthy rats. (C) Immunostaining of DDAH-2 (B-b, d) in the kidney of healthy rats (control of specificity of anti-DDAH-2). The specificity of the secondary antibody was verified by incubating kidney sections without anti-DDAH-2 (B-a, c). Number of animals, n = 3. The microscope used was a Nikon Eclipse E400 equipped with Sony DXC-390P camera (objective: X20, X40). Abbreviations: DDAH-2, N^G^, N^G^-dimethylarginine dimethylaminohydrolase isoform 2; see also figure 2.

For DDAH-2, a cytosolic immunoreactivity was observed in the neurons of submedial (Sub), mediodorsal (MD), ventral posterolateral (VPL), posteromedial (VPM), laterodorsal (LD), paracentral (PC), posterior (Po), ventromedial (VM), ventrolateral (VL), and centrolateral (CL) thalamic nuclei (Fig. 4A). Unlike DDAH-1 and in agreement with western blot data (Fig. 2 B), our immunohistochemical study revealed an increase in DDAH-2 protein expression in infected rats in comparison to non-infected animals (see the example of the ventral and posterior part of the thalamus in Fig. 4B).

The specificity of anti-DDAH-1 and anti-DDAH-2 antibodies used in the brain was verified by deploying the same immunohistochemical procedure in a rat kidney. DDAH was, indeed, predominantly expressed in this organ (Fig. 3B). For DDAH-1, the Immunostaining reactivity was exhibited in the cytoplasm of the proximal convoluted tubular cells (Fig. 3B-b, d). For DDAH2, the immunostaining was localized in the distal convoluted tubular cells and the cortical and inner medullary collecting ducts (Fig. 4C-b, d). A lack in staining was observed when either the anti-DDAH-1 or anti-DDAH-2 antibody were omitted in the immunohistochemical procedure (Fig. 3B-a, c and 4C-a, c).

#### Morphometric analysis of DDAH-1 and DDAH-2 diencephalic expressing cells

In the ventral and posterior parts of the thalamus, a Fisher's post-hoc analysis revealed that, in infected rats, the surface area of the soma and perimeter of cells expressing DDAH-2 were significantly larger *versus* those expressing DDAH-1 (Table 3). These two groups of cells exhibited, however, a very close form factor (FF) (Table 3). Considered as a whole, the analyses confirmed that DDAH-1 expressing cells belong to the interneuron cellular type.

**Table 3:** Morphometric parameters for differentiation of DDAH-1 and DDAH-2 cell groups in *Trypanosoma brucei brucei*-infected Wistar rats.

	Soma area (µm^2^)	Soma perimeter (µm)	Soma FF
**DDAH-1 (** ***n = 134*** **)**	33.33±0.61	26.01±0.36	0.63±0.01
**DDAH-2 (** ***n = 134*** **)**	125.51±3.35	52.80±0.94	0,57±0,09
***P*** ** value** *	<0.0001	<0.0001	N.S**

*The values given represent the mean ± standard deviation; a Fisher's post-hoc analysis was achieved.

FF: The form factor.

**N.S: Non significant.

### Expression of the two arginase isoforms

Since both isoforms of arginase are expressed in the brain, we tried to confirm the lack of differences in arginase activity in the diencephalic brain structures (hypothalamus/thalamus) between control and infected rats. In this respect, protein (western blot) and mRNA (real-time RT-PCR) levels of arginase-1 and arginase-2 were analyzed in diencephalic brain structures during the time course of the infection.

#### Western blot analysis of arginase-1 and arginase-2

In agreement with data on arginase activity, arginase-1 and arginase-2 protein levels did not differ between control and infected rats throughout the time course of the infection (Fig. 2E).

#### Analysis of the mRNA of arginase-1 and arginase-2

As expected from data on arginase activity and western blot analysis, no significant changes were observed in diencephalic brain structures for mRNA transcripts of arginase-1 and arginase-2 (Fig. 2F).

### Changes occurring in the amino acids content throughout the time course of the disease

**Figure 5:**
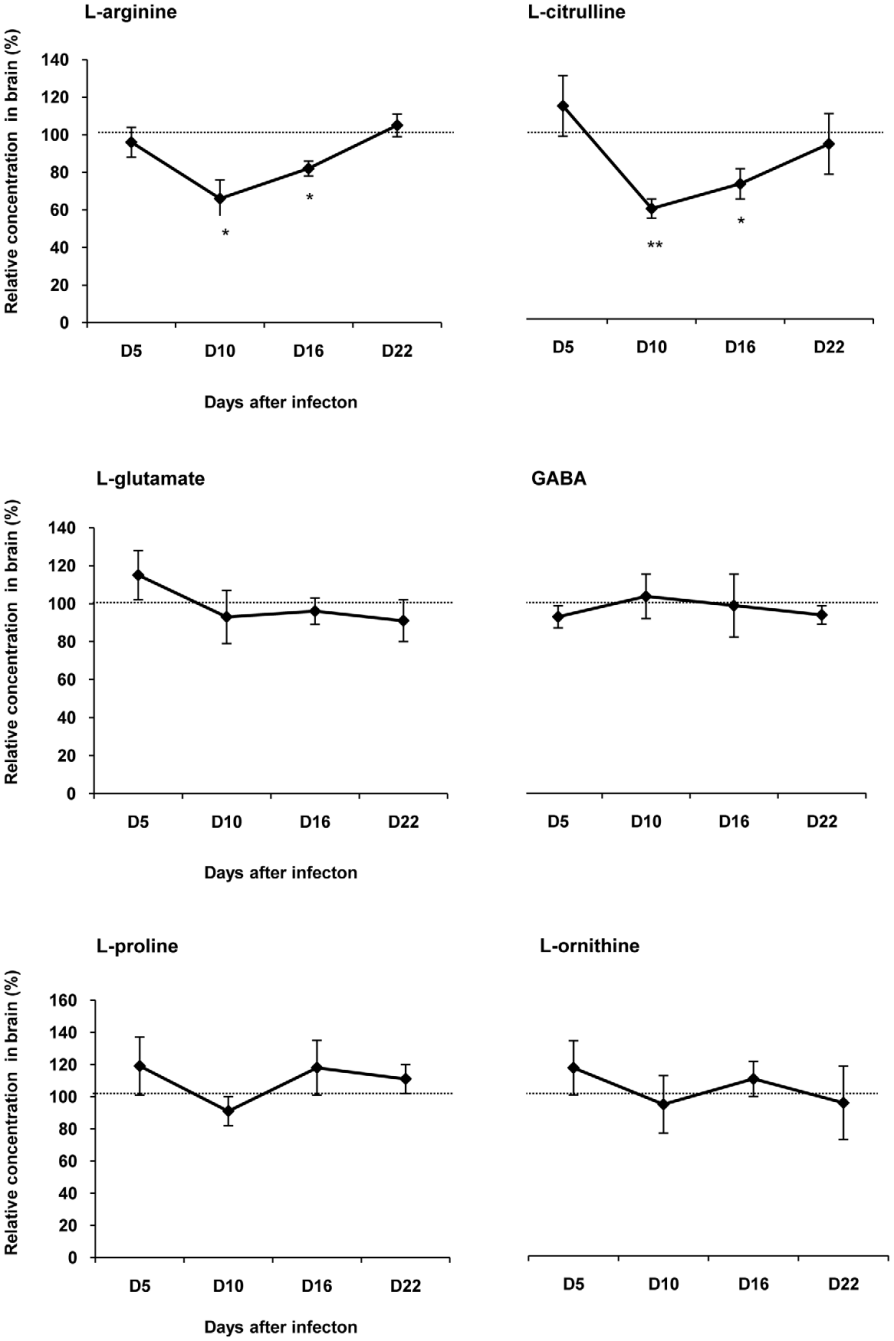
Changes occurring in the amino acid content in the brain during the course of infection in *T. b. b.*-infected rats. The variations of the concentration of amino acids (mean value ± SEM) in the diencephalon (hypothalamus/thalamus) of infected rats were normalized with the corresponding values obtained in healthy control animals. 100% of the control value (delineated by the dotted line) corresponds to: 34±2 µM for L-arginine; 10±1 µM for L-citrulline; 892±36 µM for L-glutamate; 796±80 µM for L-GABA; 16±1 µM for L-proline and 5±0.4 µM for L-ornithine. Concentration of ADMA was undetectable. Statistics: ANOVA followed by post-hoc Fisher's test (*p<0.05, **p<0.001 compared to healthy rats); n = 6 animals in each group at the different times post-infection: D5, D10, D16 and D22. Abbreviations: GABA, γ-aminobutyric acid; see also figure 2.

In diencephalic brain structures, the main amino acids involved in the metabolic pathways catalyzed by NOS, DDAH and arginase were quantified from D5 to D22 after infection. In infected rats, the variations of relative concentrations of L-arginine and L-citrulline were in the same range. Compared to healthy control rats, their concentrations decreased significantly at D10 (L-arginine, 65.87±17.07%, p<0.05; L-citrulline, 57.76±5.03%, p<0.001; n = 6) and D16 (L-arginine, 82.33±4.39%, p<0.05; L-citrulline, 70.52±8.17%, p<0.05; n = 6). At D22 post infection, the relative concentrations of the two amino acids returned to control values (Fig. 5). No significant change was observed in the relative concentrations of L-glutamate, L-glutamine (data not shown), GABA, L-proline and L-ornithine in infected rats throughout the investigation (Fig. 5). The presence of ADMA in diencephalic brain structures was not detectable (data not shown).

**Figure 6:**
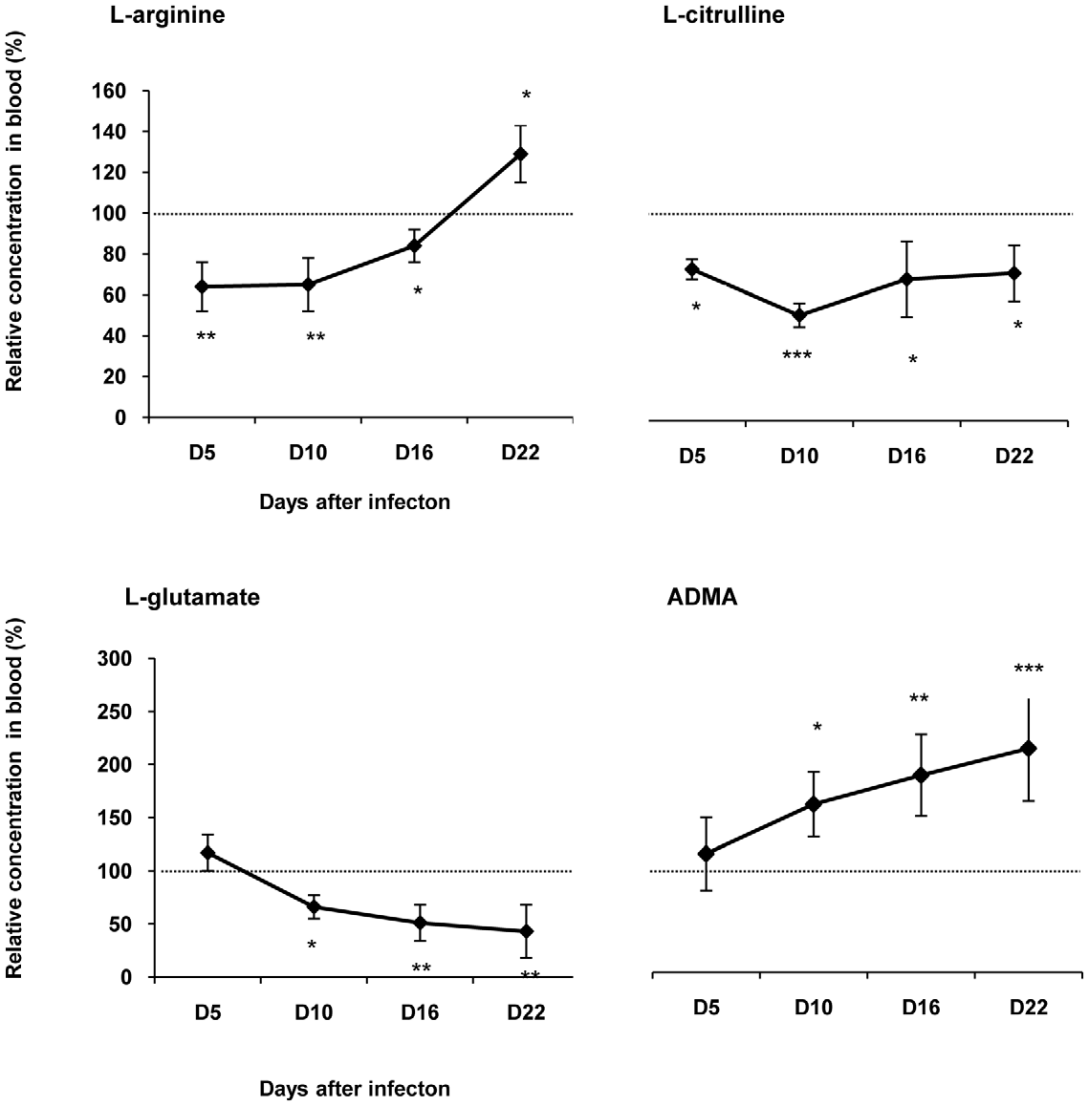
Relative changes in the content of amino acid in blood during the course of infection in *T. b. b.*-infected rats. The changes in the concentration of amino acids (mean value ± SEM) in the blood of infected rats were normalized with the corresponding values obtained in healthy control animals. 100% of the control values (delineated by the dotted line) correspond to: 110±3 µM for L-arginine; 100±4 µM for L-citrulline; 95±4 µM for L-glutamate and 0.96±0.1 µM for ADMA. Statistics: ANOVA followed by post-hoc Fisher's test (*p<0.05, **p<0.001, ***p<0.0001 compared to healthy rats); n = 6 animals in each group at the different times post-infection: D5, D10, D16 and D22. Abbreviations: ADMA, asymmetric dimethylarginine; see also figure 2.

Amino acids concentrations were also analyzed in the blood. In infected rats, the relative concentrations of L-arginine and L-citrulline decreased significantly at D5, D10 and D16 (Fig. 6). At D22, the concentration of L-citrulline remained lower than control values (73.87±13.67%, p<0.05, n = 6), while that of L-arginine increased slightly above control values (128.76±14.37%, p<0.05) (Fig. 6). L-glutamate (Fig. 6) and L-glutamine (data not shown) relative concentrations decreased significantly, from D10 (L-glutamate, 66.44±10.61%, p<0.05; L-glutamine, 60.06±13.10%, p<0.05; n = 6) to D16 (L-glutamate, 51.15±17.45%, p<0.001; L-glutamine, 33.25±16.71%, p<0.001; n = 6), and D22 (L-glutamate, 43.24±24.55%, p<0.001; L-glutamine, 18.90±4.04%, p = 0.0001; n = 6). Plasma relative concentration of ADMA increased significantly throughout the time course of the illness, from D10 (155.96±30.44%, p<0.05, n = 6) to D16 (182.51±37.99%, p<0.001, n = 6), and D22 (207.91±48.58%, p<0.001, n = 6), (Fig. 6).

## Discussion

First of all, we must emphasize the adequacy of the *T. b. brucei*-infected rat model in mimicking closely the two-stage disease in human *T. b. gambiense* infection. As previously reported in our rat model, the initiation of the neurological stage of the disease is characterized by a marked decrease in body weight gain and the presence of trypanosomes in the CSF 10 days post-infection [2]. The neurological stage is also marked by an increase in cerebral iNOS activity, leading to an overproduction of NO in the brain. By use of double labeling, we have also shown that the iNOS immunoreactivity is located in glial elements (microglia and astrocytes) as well as in neurons, especially in brain structures involved in the sleep/wake regulation (lateral and posterior part of the hypothalamus, perifornical area) [2]. The present investigation was designed to study the molecular changes occurring in arginase and DDAH in the brain at the key stages of the HAT infection. We suggest that DDAH most likely contributes to the regulation of iNOS activity in the brain, whereas arginase has a slight effect, if any, contrary to its role in the periphery.

### Arginase pathway

Concerning the arginase pathway, experimental data obtained in the brain indicate that, throughout the time course of the disease, no significant changes occurred in the total activity of arginase in the protein or in the mRNA levels of the two isoforms of the enzyme (arginase-1 and -2). This is also evident from the absence of any significant changes in the concentration of the major amino acids involved in the arginase pathway, such as L-ornithine, L-proline, L-glutamate, L-glutamine, and L-GABA. Since the arginase activity remained unchanged throughout the course of the experimental illness, the decrease in L-arginine content, observed at D10 and D16 post-infection, may be due to the utilization of the substrate through the iNOS pathway [2]. Nevertheless, we cannot exclude a possible increase in the arginosuccinate synthetase (ASS) and arginosuccinate lyase (ASL) activities which would favor an increase in the concentration of L-arginine by recycling L-citrulline to L-arginine. Such a mechanism has been reported in rats subjected to brain excitotoxicity mediated by kainic acid. An increase in the production of NO in parallel to ASS and ASL activities takes place in the cerebral cortex, cerebellum and brain stem, while the arginase activity remains unchanged [18]. These mechanisms remain to be further investigated.

In the present approach, we did not examine the changes occurring in arginase activity in the periphery, as such changes have already been addressed [4], [11]. In this respect, we suggested [2] that arginase activity changes occurring in the periphery may reflect the strategy developed by trypanosomes to invade the host and insure their own survival and development through (i) enhancement of arginase activity mediating the synthesis of polyamines necessary to trypanosome growth, and (ii) limitation of trypanocidal properties carried out by the decrease in NO production. Arginase appears therefore to be a critical regulator of NO synthesis, through a competition with iNOS for L-arginine [19]. In this respect, a significant decrease in plasma L-arginine concentration was observed in our infected rats. Such a change was also reported in *T. b. brucei*-infected mice [4] and *T. b. gambiense*-infected voles [20]. A large proportion of the L-arginine in the blood might be metabolized by arginase since iNOS is inhibited during the time course of the infection [2], reducing NO production and, consequently, trypanocidal pressure. In support of this interpretation, L-arginine supplementation in *T. b. brucei*-infected mice restores both NO production and NO trypanocidal activity [4], [11]. Furthermore, in the periphery, the arginase genes are up-regulated throughout the course of the infection. Arginase-1 and, to a lower extent, arginase-2 genes can be induced directly by the trypanosomes. Moreover, interleukin-4 (IL-4), IL-10 and Transforming Growth Factor-β (TGF-β), which are potent arginase inducers, are enhanced in experimental trypanosomiasis [21]. Alternatively, arginase activation can also result from a direct interaction between trypanosome products, such as the trypanosome-derived lymphocyte-triggering factor (TLTF), and T lymphocytes [22]. In any case, arginase activation enhances the production of polyamines that are essential for the synthesis of the trypanothione, a compound necessary for growth and survival of trypanosomes (Fig. 1). Trypanothione synthesis from glutathione and spermidine [23] may account for the significant decrease in glutamate and glutamine concentration observed at the peripheral level in our infected rats.

In the brain, the situation does not appear to be favorable for trypanosome multiplication and spread, since trypanocidal NO increases and arginase activity (necessary for trypanosome growth) remain stable. These mechanisms may act as additive protective elements for the brain, although trypanosomes will still be able to enter the central nervous system. The parasites pass through the blood-brain barrier (BBB) across or between the endothelial cells and the vessel basement membranes [24]. The trypanosomes are present in cerebrospinal fluid (CSF), especially in such areas where the BBB is weak, i.e., at the level of the circumventricular organ and around the ventricular cavities [2], [25], [26]. Due to the large concentration of NO in the extracellular space of the brain parenchyma [2], it is likely that trypanosomes cannot penetrate easily into deeper structures. Furthermore, trypanosomes present in the brain parenchyma are described as being damaged [27], underlining potential difficulties to survive in such conditions. Intracellular localization of African trypanosomes may also protect the parasites against the trypanocidal properties of NO [28], [29]. Due to the lack of changes observed in the arginase activity during the course of the infection, the energy necessary for trypanosome growth may be supplied by the high rate of the brain metabolism [30].

### DDAH/ADMA pathway

Similar to our observations on the arginase pathway, the DDAH/ADMA pathway may also play a key role in the regulation of NO production since ADMA acts as a potent iNOS inhibitor [31]. Indeed, we demonstrate that the cerebral activity of DDAH, mRNA and protein expression increase significantly in the later stage of the disease (at D10 and D16 after infection) concomitant with the activation of iNOS (activity and protein expression) and the enhancement of NO production [2]. The tight correlation existing between these events supports the hypothesis of a contribution of DDAH in the control of NO production (particularly at D16) in the brain during the invasion by trypanosomes. In line with this view, it was suggested that, in inflammatory conditions (such as in the present case), the activation of DDAH may contribute to reduce the level of ADMA [32].There are also reports indicating that ADMA concentration is significantly lower (−48%) in the CSF of patients suffering of Alzheimer's disease or in pathological aging with cognitive impairment [33]. Trypanosomiasis might thus also contain insidious neurodegenerative processes and therefore represent a model of neurodegenerative diseases.

In our experimental model, the cerebral increase in DDAH activity is mainly dependent on the DDAH-2 isoform which is transcriptionally regulated, a specific increase in DDAH-2 mRNA occurring throughout the infection. The DDAH-2 gene is located on chromosome 6 (6p21.3) in a region containing various genes involved in inflammatory processes linked with the autoimmune disease susceptibility [9]. This localization and the wide expression of DDAH-2 in immune cells suggest that the DDAH-2 gene may represent a potential disease-susceptibility gene. In this sense, it has been reported that inflammatory cytokines such as IL-1β are capable of simulating simultaneously iNOS and DDAH in vascular smooth muscle cells [34]. This effect is accompanied by an increase in NO metabolites and a decrease in ADMA content in culture media. A chronic overexpression of proinflammatory cytokines (IL-1, TNF-α) and IFN-γ in the brains of *T. b.* -infected rats, might be responsible for the iNOS and DDAH-2 up-regulation, leading to a reduction of trypanosome proliferation, neuroinflammation and neurodegeneration [35].

In diencephalic brain structures, DDAH-2 immunoreactivity was primarily observed in neurons. It was enhanced in infected rats as compared with healthy animals. Contrary to DDAH-2, no significant change was noticed with DDAH-1 immunoreactivity between infected and non-infected animals. It should be noted that DDAH-1 immunoreactivity is located in interneurons which express also nNOS [36], [37], an enzyme that remains unchanged throughout the *T. b. b.* infection process [2].

Recently, Gow et al. [38] reported that NO may S-nitrosylate a distinct subset of cellular proteins, DDAH being one of them. The cellular proteins may be regulated by S-nitrosylation through the NO derived from iNOS [10]. Under certain conditions, when NO increases, the S-nitrosylation of DDAH decreases its activity. This mechanism would lead to an accumulation of ADMA and consequently to the inhibition of the iNOS. The S-nitrosylation of DDAH provides thus a potential feedback mechanism for the regulation of NO production. This mechanism could explain why, in our experimental model of HAT, the activities of both iNOS and DDAH return simultaneously to the control level at D22 after infection. Such an effect may reflect the *pre-mortem* situation of our infected animals at that time of the infection course. In such extreme conditions, anti-inflammatory processes may be triggered to protect the brain as reported in autoimmune encephalitis [39].

Peripheral ADMA concentration was elevated in our *T. b. brucei-*infected rats. These changes coincide with the entry into the second stage of the disease and correlate with the degree of the infection. The mechanism responsible for the increase in the ADMA level was not specifically investigated in this study. It may be explained, at least partly, by the existence of a decrease in the concentration of L-citrulline which occurs in parallel with the ADMA increase. It is also known that ADMA accumulation is associated with an endothelial dysfunction [40]. Recent *in vitro* and *in vivo* studies indicate that an enhanced ADMA synthesis coincides with an increased activity of the immune pathways, such as in HIV infected patients [41], [42]. In this respect, ADMA can be produced by the endothelial cells and the activated mononuclear blood cells [42]. The latter cellular elements release simultaneously Th1-type cytokine, interferon-γ (IFN-γ, an important NOS inducer) and ADMA [41]. It has also been reported that even modest changes in the ADMA level can have significant effects on NO synthesis [31]. In our previous studies [2], we reported that circulating NO concentration decreases significantly from D10 post-infection (in correlation with illness progression) and that this decrease is likely to depend on an impaired iNOS activity in macrophages. We suggest that ADMA may also contribute to the inhibition of NO synthesis in the periphery.

#### Conclusion

**Figure 7:**
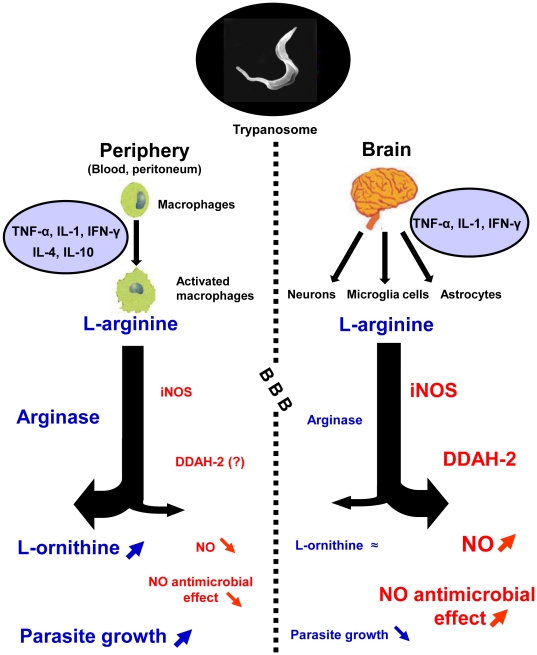
Schematic representation of the enzymatic pathways involved in the control of trypanosome entry into the brain during the later stage of the disease. In the periphery (blood), the arginase is strongly expressed in the activated macrophages and competes with iNOS for a common substrate, L-arginine. This phenomenon, under control of cytokines, leads to a decrease in iNOS activity and consequently in the production of NO, a gaseous messenger possessing trypanocidal properties. Such a mechanism, favoring the growth and multiplication of the parasites, is strengthened by the accumulation of the endogenous inhibitor of iNOS, ADMA, as observed in the blood. An opposite mechanism is observed in the brain since the activity of iNOS increases in the three cell types: neurons, microglia and astrocytes. DDAH-2 is over expressed in neurons, likely by decreasing the ADMA pool, and may enhance iNOS activity and, consequently, NO production. Concomitantly, the activity and expression of the arginase remain unchanged. Abbreviations: NO, nitric oxide; iNOS, inducible nitric oxide synthase; ADMA, asymmetric dimethylarginine; DDAH, N^G^, N^G^-dimethylarginine dimethylaminohydrolase. Small arrows indicate an increase in the given product when pointing upwards and a decrease when pointing downwards. Hyphen is used to indicate that the amount in the given molecule is stable.

In the *T. b. brucei-*infected rat model of human African trypanosomiasis, we demonstrated that the activity of cerebral arginase remains constant throughout the time course of the infection. Simultaneously, DDAH-2 isoform increased, contributing to an overproduction of NO, undoubtedly through a reduction of the ADMA level, a known iNOS inhibitor. These changes differed from those reported in the periphery in the same animal model (Fig. 7). They may constitute an additive protection against the trypanosomes that have entered the central nervous system, through the increase in the trypanocidal pressure carried by NO, while the arginase activity necessary for trypanosomes growth remained unchanged. The involvement of NO overproduction in the pathophysiology of the neurological stage of trypanosomiasis might also be considered.

## References

[1] Buguet A, Bourdon L, Bouteille B, Cespuglio R, Vincendeau P (2001). The duality of sleeping sickness: focusing on sleep.. Sleep Med Rev.

[2] Amrouni D, Gautier-Sauvigné S, Meiller A, Vincendeau Ph, Bouteille B (2010). Cerebral and peripheral changes occurring in nitric oxide (NO) synthesis in a rat model of sleeping sickness: Identification of brain iNOS expressing cells.. PloS One.

[3] Alderton WK, Cooper CE, Knowles RG (2001). Nitric oxide synthases: structure, function and inhibition.. Biochem J.

[4] Gobert AP, Daulouede S, Lepoivre M, Boucher JL, Bouteille B (2000). L-arginine availability modulates local nitric oxide production and parasite killing in experimental trypanososmiasis.. Infect Immun.

[5] Antoine-Moussiaux N, Magez S, Desmecht D (2008). Contributions of experimental mouse models to the understanding of African trypanosomiasis.. Trends Parasitol.

[6] Yu H, Iyer RK, Kern RM, Rodriguez WI, Grody WW (2001). Expression of arginase isoforms in mouse brain.. J Neurosci Res.

[7] Hecker M, Nematollahdi H, Hey C, Busse R, Racké K (1995). Inhibition of arginase by N-hydroxy-L-arginine in alveolar macrophages: implications for the utilization of L-arginine for nitric oxide synthesis.. FEBS Lett.

[8] Tsikas D, Böger RH, Sandmann J, Bode-Böger SM, Frölich JC (2000). Endogenous nitric oxide synthase inhibitors are responsible for the L-arginine paradox.. FEBS Lett.

[9] Tran CT, Fox MF, Vallance P, Leiper JM (2000). Chromosomal localization, gene structure, and expression pattern of DDAH1: Comparison with DDAH2 and implications for evolutionary origins.. Genomics.

[10] Leiper J, Murray-Rust J, McDonald N, Vallance P (2002). S-nitrosylation of dimethylarginine dimethylaminohydrolase regulates enzyme activity: Further interactions between nitric oxide synthase and dimethylarginine dimethylaminohydrolase.. Proc Natl Acad Sci U S A.

[11] Duleu S, Vincendeau P, Courtois P, Semballa S, Lagroye I (2004). Mouse strain susceptibility to trypanosome infection: An arginase-dependent effect.. J Immunol.

[12] Bradford MM (1976). A rapid and sensitive method for the quantification of microgram quantities of protein utilizing the principle of protein-dye binding.. Anal Biochem.

[13] Liu P, Smith PF, Appleton I, Darlington CL, Bilkey DK (2003). Nitric oxide synthase and arginase in the rat hippocampus and the entorhinal, perirhinal, postrhinal, and temporal cortices: Regional variations and age-related changes.. Hippocampus.

[14] Prescott LM, Jones ME (1969). Modified Methods for the determination of carbamyl aspartate.. Anal Biochem.

[15] Meiller A, Alvarez S, Drané P, Lallemand C, Blanchard B (2007). p53-dependent stimulation of redox-related genes in the lymphoid organs of gamma-irradiated mice: identification of haeme-oxygenase 1 as a direct p53 target gene.. Nucleic Acids Res.

[16] Piraud M, Vianey-Saban C, Bourdin C, Acquaviva-Bourdain S, Boyer S (2005). A new reversed-phase liquid chromatographic/tandem mass spectrometric method for analysis of underivatised amino acids: evaluation for the diagnosis and the management of inherited disorders of amino acids metabolism. Rapid commun.. Mass Spectrom.

[17] Schwedhelm E, Tan-Andrese J, Maas R, Riederer U, Schulze F (2005). Liquid chromatography-tandem mass spectrometry method for the analysis of asymmetric dimethylarginine in human plasma.. Clin Chem.

[18] Swamy M, Sirajudeen KN, Chandran G (2009). Nitric oxide (NO), citrulline-NO cycle enzymes, glutamine synthetase, and oxidative status in kainic acid-mediated excitotoxicity in rat brain.. Drug Chem Toxicol.

[19] Durante W, Johnson FK, Johnson RA (2007). Arginase: a critical regulator of nitric oxide synthesis and vascular function.. Clin Exp Pharmacol Physiol.

[20] Newport GR, Page CR, Ashman PU, Stibbs HH, Seed JR (1977). Alteration of free serum amino acids in voles infected with *Trypanosoma brucei gambiense*.. J Parasitol.

[21] Uzonna JE, Kaushik RS, Gordon JR, Tabel H (1999). Cytokines and antibody response during *Trypanosoma congolense* infections in two inbred mouse strains that differ in resistance.. Parasite Immunol.

[22] De Baetselier P, Namangala B, Noël W, Brys L, Pays E (2001). Alternative versus classical macrophage activation during experimental African trypanosomiasis.. Int J Parasitol.

[23] Müller S, Liebau E, Walter RD, Kraut-Siegel RL (2003). Thiol-based redox metabolism of protozoan parasites.. Trends Parasitol.

[24] Masocha W, Rottenberg ME, Kristensson K (2007). Migration of African trypanosomes across the blood-brain barrier.. Physiol Behav.

[25] Abolarin MO, Evan DA, Tovey DG, Ormerod WE (1982). Cryptic stage of sleeping-sickness trypanosome developing in choroid plexus epithelial cells.. Br Med J.

[26] Ormerod WE, Venkatesan S (1971). The occult visceral phase of mammalian trypanosomes with special reference to the life cycle of *Trypanosoma* (Trypanozoon) *brucei*.. Trans R Soc Trop Med Hyg.

[27] Van Marck EA, Le Ray D, Beckers A, Jacob W, Wery M (1981). Light and electron microscope studies on extracellular *Trypanosoma brucei gambiense* in the brain of chronically infected rodents.. Ann Soc Belg Med Trop.

[28] Mattern P, Mayer G, Felici M (1972). Existence of amastigote forms of *Trypanosoma gambiense* in the choroidal plexal tissue of experimentally infected mice.. C R Acad Sci D.

[29] Stoppini L, Buchs PA, Brun R, Muller D, Duport S (2000). Infection of organotypic slice cultures from rat central nervous tissue with *Trypanosoma brucei brucei*.. Int J Med Microbiol.

[30] Cespuglio R, Colas D, Gautier-Sauvigné S, Velluti R, Parmeggiani PL (2005). Energy processes underlying the sleep-wake cycle.. The physiological nature of sleep.

[31] Dayoub H, Achan V, Adimoolam S, Jacobi J, Stuehlinger MC (2003). Dimethylarginine dimethyaminohydrolase regulates nitric oxide synthesis: genetic and physiological evidence.. Circulation.

[32] Tran CT, Leiper JM, Vallance P (2003). The DDAH/ADMA/NOS pathway.. Atherosclerosis.

[33] Abe T, Tohgi H, Murata T, Isobe C, Sato C (2001). Reduction in asymmetrical dimethylarginine, an endogenous nitric oxide synthase inhibitor, in the cerebrospinal fluid during aging and in patients with Alzheimer's disease.. Neurosci Lett.

[34] Ueda S, Kato S, Matsuoka H, Kimoto M, Okuda S (2003). Regulation of cytokine-induced nitric oxide synthesis by asymmetric dimethylarginine: Role of dimethylarginine dimehtylaminohydrolase.. Circ Res.

[35] Quan N, Mhlanga JDM, Whiteside MB, Mccoy AN, Kristensson K (1999). Chronic overexpression of proinflammatory cytokines and histopathology in the brains of rats infected with *Trypanosoma brucei*.. J Comp Neurol.

[36] Bertini G, Peng ZC, Bentivoglio M (1996). The chemical heterogeneity of cortical interneurons: Nitric oxide synthase vs. calbindin and paravalbumin immunoreactivity in the rat.. Brain Res Bull.

[37] Koliatsos VE, Kecojevic A, Troncoso JC, Gastard MC, Bennett DA (2006). Early involvement of small inhibitory cortical interneurons in Alzheimer's disease.. Acta Neuropathol.

[38] Gow AJ, Chen Q, Hess DT, Day BJ, Ischiropoulos H (2002). Basal and stimulated protein S-nitrosylation in multiple cell types and tissues.. J Biol Chem.

[39] Cua DJ, Hutchins B, Laface DM, Stohlman SA, Coffman RL (2001). Central nervous system expression of IL-10 inhibits autoimmune encephalomyelitis.. J Immunol.

[40] Fliser D (2005). Asymmetric dimethylarginine (ADMA): the silent transition from an ‘uraemic toxin’ to a global cardiovascular risk molecule.. Eur J Clin Iinvest.

[41] Schroecksnadel K, Weiss G, Stanger O, Teerlinks T, Fuchs D (2007). Increased Asymmetric dimethylarginine concentrations in stimulated peripheral blood mononuclear.. Scand J Immunol.

[42] Kurz K, Teerlink T, Sarcletti M, Weiss G, Zangerle R (2009). Plasma concentrations of the cardiovascular risk factor asymmetric dimethylarginine (ADMA) are increased in patients with HIV-1 infection and correlate with immune activation markers.. Pharmacol Res.

